# Antioxidant and Anticancer Activities of Essential Oil from Gannan Navel Orange Peel

**DOI:** 10.3390/molecules22081391

**Published:** 2017-08-22

**Authors:** Chao Yang, Hui Chen, Hongli Chen, Balian Zhong, Xuzhong Luo, Jiong Chun

**Affiliations:** National Navel Orange Engineering Research Center, College of Life and Environmental Sciences, Gannan Normal University, Ganzhou 34100, China; yac508@126.com (C.Y.); chenhui0910102@163.com (H.C.); mxj886@163.com (H.C.); bal.zh@163.com (B.Z.); luoxuzhong@hotmail.com (X.L.)

**Keywords:** Gannan navel orange, essential oil, GC-MS, anticancer

## Abstract

China is one of the leading producers of citrus in the world. Gannan in Jiangxi Province is the top navel orange producing area in China. In the present study, an essential oil was prepared by cold pressing of Gannan navel orange peel followed by molecular distillation. Its chemical composition was analyzed by GC-MS. Twenty four constituents were identified, representing 97.9% of the total oil. The predominant constituent was limonene (74.6%). The anticancer activities of this orange essential oil, as well as some of its major constituents, were investigated by MTT assay. This essential oil showed a positive effect on the inhibition of the proliferation of a human lung cancer cell line A549 and prostate cancer cell line 22RV-1. Some of the oil constituents displayed high anticancer potential and deserve further study.

## 1. Introduction

Cancer is a group of diseases that endanger human life and currently it has become the first leading cause of death in the world. There were an estimated 14.1 million new cases of cancer and 8.2 million deaths from cancer in 2012 [1]. Cancer cells have characteristics of uncontrolled growth and invasion, thus effective inhibition of cancer proliferation is a method for cancer therapy [2]. Chemoprevention is the hot spot and the leading edge of cancer prevention. It is a method to prevent or slow the development of cancer by using chemical or biological substances, thereby reducing the incidence and mortality of cancer [3]. Previous studies have revealed that oxidative stress played a key role in the etiology of a wide array of human diseases, including cancer [4]. Natural or synthetic antioxidants have been studied with respect to their protective effect against free radical damage that may be the cause of cancer. Natural products, such as paclitaxel, camptothecin, vinblastine, and matrine, have been used as chemoprophylactic drugs and proved to be effective in the treatment of cancer [5,6,7]. So far, the anticancer activities of essential oils from extraction of plants have attracted much interest. Essential oils are a diverse group of natural products that are composed of terpene hydrocarbons, alcohols, aldehydes, ketones, and esters [8,9]. Monoterpenes are the major components of essential oils in the extracts from many plants.

Citrus fruits, the generic collective name for oranges, mandarins, limes, lemons, grapefruits and citrons, belong to the genus *Citrus* of the family Rutaceae. Navel orange is a type of citrus which is among the most popular orange varieties. It has good economic value and is widely cultivated around the world, in countries such as China, Brazil, the United States, and Spain. Gannan in Jiangxi Province is the top navel orange producing area in China, with an area of 0.11 million hectares and a total annual output of 1.2 million tons. Previous studies indicated that navel oranges contain many useful natural products such as essential oils, flavonoids, carotenoids and vitamins [10,11]. Exploring and exploiting citrus essential oil is an additional way to increase the economic value of the crop due to its special roles in the food, flavor and cosmetics industries. Citrus essential oils and their components have also drawn much attention as chemoprevention agents in cancer treatment. Palestine sweet lime essential oil could inhibit inflammation and activate apoptosis of human SW480 colon cancer cells by suppressing the expression of both COX-2 and IL-6 [12]. d-Limonene, a major constituent of citrus essential oil, is recognized as a potential chemotherapeutic agent because it can induce human colon cancer cell apoptosis via the mitochondrial death pathway and suppress the PI3K/Akt pathway [13]. Perillyl alcohol, an oxygenated monoterpene constituent of citrus essential oil, has a good effect in clinical treatment of patients with malignant brain tumors [14]. Blood orange essential oil could inhibit angiogenesis, metastasis and cell death in human colon cancer cells [15]. However, the anticancer activity of navel orange essential oil has not been reported in the literature.

The current method used in most countries for manufacturing cold-pressed orange oil is simultaneous extraction of a juice and oil emulsion from the whole fruit. Cold-Pressed orange oil contains waxes, pesticide residues and carotenoids, which may interfere with our anticancer activity research. To avoid this problem, we used a molecular distillation method to remove them. The separation principle of molecular distillation is based on the difference of molecular mean free path [16]. It is very suitable for heat sensitive compounds and characterized by very high vacuum so that the product can be obtained at low temperature [17,18]. Although chemical components of most orange essential oils have been identified, their anticancer activities have not been investigated systematically. In this study, we prepared an essential oil sample (Navel Orange Essential Oil, NOEO) by molecular distillation of cold-pressed navel orange oil. Its constituents were identified by GC-MS. An MTT assay was used to evaluate the effects of this orange oil sample and its seven single constituents on the proliferation of a human lung cancer cell line A549 and prostate cancer cell line 22RV-1. We expect this work will provide information for developing chemopreventive agents for cancer treatment.

## 2. Results

### 2.1. Chemical Composition of the Essential Oil of Navel Orange

**Figure 1:**
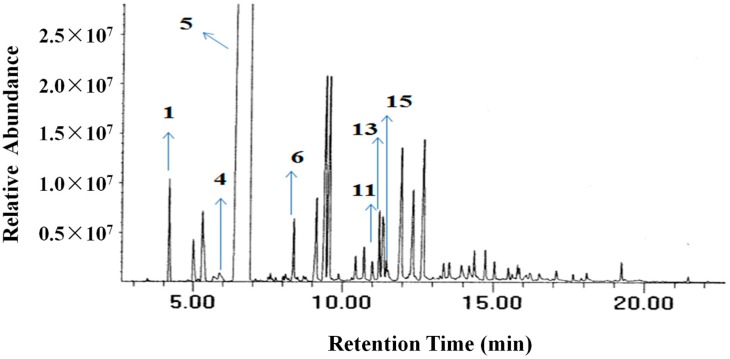
Total ion chromatogram of navel orange essential oil (NOEO).

Chemical composition of the NOEO was analyzed by GC-MS (Table 1). Figure 1 shows the total ion chromatogram (TIC) of NOEO. We selected seven single constituents to test their anticancer activity, which are marked in the TIC (**1**: α-pinene; **4**: 3-carene; **5**: limonene; **6**: linalool; **11**: citral; **13**: α-terpineol; **15**: decanal; the amount of each constituent is given in Table 1).

As shown in Table 1, twenty four compounds, accounting for 97.9% of the total oil, were identified based on their mass spectra. The main components were terpenic molecules, and the oil was composed of 97.3% monoterpenes (which include 19.0% oxygenated monoterpenes) and 0.5% sesquiterpenes. The main constituents were limonene (74.6%), limonene 1,2-epoxide (3.5%), *cis*-*p*-mentha-2,8-dien-1-ol (3.2%) and (*E*)-carveol (2.4%). Because of the ability to stabilize the symptoms of chronic diseases including cancer, monoterpenes are gaining more attention as constituents of prophylactic formulations [19]. Monoterpenes derived from citrus have been shown to inhibit human cancer cell proliferation and tumor growth through various mechanisms such as promoting cell apoptosis, inhibition of the expression of growth factors and cell cycle arrest [20].

### 2.2. Antioxidant Activity

There are many kinds of oxidants in our foods. These oxidants can induce some diseases, such as hypertension, diabetes, arteriosclerosis, cancer and senescence [21,22]. Synthetic antioxidants can solve this problem, but they have risks and side effects on humans. Thus, antioxidants from plants are attracting much attention.

The 2,2-diphenyl-1-picrylhydrazyl (DPPH) and 2,2′-azino-bis(3-ethylbenzthiazoline-6-sulfonic acid (ABTS) assays are the classic methods to detect the antioxidant activity of chemical compounds. The antioxidant activity of the NOEO was therefore measured according to the DPPH assay and ABTS assay, with IC_50_ values of 2.19 ± 0.20 mg/mL and 2.00 ± 0.19 mg/mL, respectively. Our results showed that NOEO had better antioxidant activity than cold-pressed orange oil in the DPPH assay (IC_50_ = 3.01 ± 0.20 mg/mL) and in the ABTS assay (IC_50_ = 23.25 ± 0.84 mg/mL) [23]. NOEO also had better antioxidant activity than pomelo oils or cold-pressed grapefruit oil in the DPPH assay (EC_50_ > 40 mg/mL) and better activity than distilled grapefruit oil in the ABTS assay (EC_50_ = 27.5 mg/mL) [24].

### 2.3. Anticancer Activity

**Figure 2:**
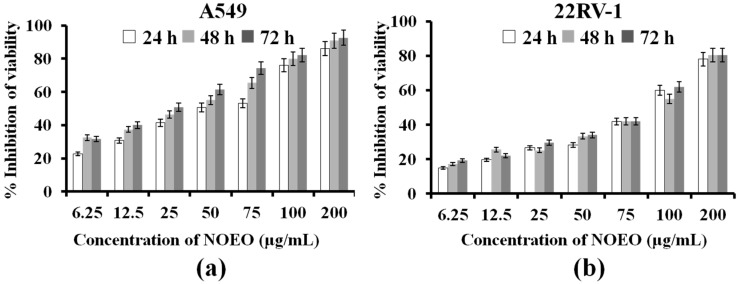
Effect of different concentrations of NOEO on viability of A549 (**a**) and 22RV-1 (**b**). Cytotoxicity was assessed by MTT assay after treatment with NOEO for 24 h, 48 h, and 72 h.

d-Limonene, the most abundant constituent of navel orange essential oil, has been shown to have anti-proliferative and apoptosis-inducing effects [25,26], thus it has been used as a chemopreventive and chemotherapeutic agent against multiple types of tumors [27,28]. Other components of the essential oil, such as α-pinene, have been shown to inhibit growth of non-small-cell lung carcinoma cells [29]. Citral could reduce the proliferation of MDA-MB-231 cells by inhibiting the cancer stem cell marker ALDH1A3 (Aldehyde dehydrogenase 1 family, member A3) [30]. This evidence suggested that NOEO might have good anticancer activity as well. In our study, the MTT assay was used to evaluate the effect of NOEO on the cell viability of A549 (a human lung cancer) cells and 22RV1 (a human prostate cancer) cells as an assessment of anticancer activity. IC_50_ values of NOEO on A549 cells at 24 h, 48 h and 72 h were 17.53 ± 0.95, 10.70 ± 0.53, and 7.86 ± 0.38, respectively. IC_50_ values of NOEO on 22RV-1 cells at 24 h, 48 h and 72 h were 45.74 ± 1.68, 42.83 ± 1.64, and 39.79 ± 1.60, respectively. The results (Figure 2) showed that NOEO had good inhibition of proliferation of both cell lines at concentrations ranging from 6.25 to 200 µg/mL.

The inhibitory effect was dose-dependent, and the higher the concentration of NOEO, the better the inhibition. To further explore the inhibition effect of NOEO on proliferation of A549 cells, a cell apoptosis experiment was investigated. A549 cells were treated with 100 µg/mL of NOEO for 24 h and apoptotic cells were detected by Annexin-V FITC/PI assay.

**Figure 3:**
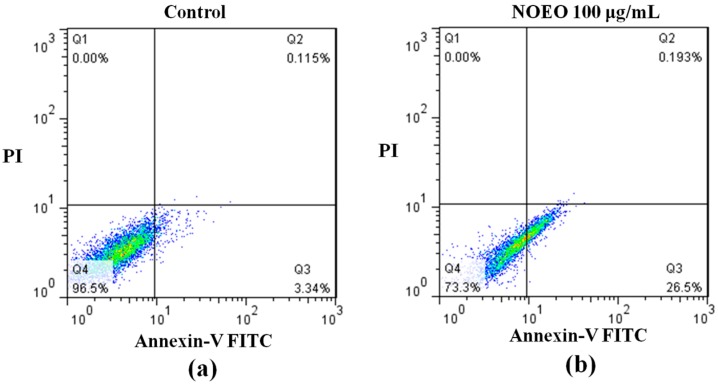
The apoptosis of A549 cells was induced by NOEO. Flow cytometric analysis of Annexin V-FITC/PI-stained A549 cells: (**a**) Cells not treated with NOEO as a control; (**b**) Cells treated with 100 μg/mL of NOEO.

As shown in Figure 3, 26.5% of the treated cells and only 3.34% of the control cells appeared to have entered early apoptosis. This result indicated that NOEO could induce apoptosis to inhibit the proliferation of A549 cells.

In an effort to explore other possible anticancer activity of the essential oil, seven of its major components were tested for their anticancer activity. NOEO is composed of terpene hydrocarbons, alcohols, aldehydes, ketones, and esters. Among them, terpene hydrocarbons, especially monoterpenes, are the predominant ingredients of NOEO. We chose three monoterpenes: limonene, which is the most predominant ingredient, α-pinene and 3-carene, which are typical ingredients of orange EO. We also chose two alcohols: linalool and α-terpineol and two aldehydes: citral and decanal to do anticancer tests because they are also important ingredients of orange EO and commercially available. As shown in Table 2, inhibition of proliferation of A549 cells were in a decreasing order as follows: linalool, 3-carene, α-terpineol, decanal, citral, d-limonene and α-pinene. Their inhibitory activities were concentration dependent (Figure 2). The IC_50_ value of the NOEO in the MTT assays was 17.53 ± 0.50 μg/mL, while those of the compounds linalool, 3-carene, α-terpineol, decanal, citral, d-limonene and α-pinene were much higher at 141.76 ± 14.59, 70.80 ± 8.24, 51.37 ± 4.92, 37.10 ± 2.96, 35.15 ± 2.38, 22.10 ± 1.94 and 22.01 ± 1.75 μg/mL, respectively. These data indicate that the capacity of the essential oil to inhibit the proliferation of A549 cells was higher than that of the seven constituents tested. Since NOEO had higher activity than any one of the seven constituents individually, it is possible that other active compounds are present or that the active components were interacting synergistically to inhibit proliferation.

Many papers have reported that citrus essential oils have anticancer activity. The essential oil of jessamine orange (*Murraya paniculata* L., Rutaceae) has cytotoxity against murine hepatoma cancer cells (Hepa 1c1c7) with an IC_50_ value of 63.7 mg/mL [31]. A dramatic increase in SH-SY5Y human neuroblastoma cell death was observed when the cultures were treated with the 0.03% of bergamot essential oil [32]. The essential oil of mandarin peel from Corrientes (Argentina) showed good cytotoxic effects against A549 cells with an IC_50_ value of 0.036 μL/mL [33]. Grapefruit and lemon essential oil also showed antitumor activities against A549 cells [34]. However, to our knowledge, the anticancer activity of navel orange essential oil has not been reported in the literature. The present study has explored the anticancer activities of some single components and the total essential oil of navel orange and might provide information for the development of more effective anticancer agents.

## 3. Discussion

Cancer cells survive by multiple molecular signaling pathways. Therefore chemopreventive agents must effectively influence different signaling pathways as compared to targeting just one pathway. Cancer chemoprevention needs the cooperation of multidisciplinary groups to find target specific and less toxic agents to block, retard, or reverse the carcinogenic process. Since citrus EOs are generally recognized as safe (GRAS) [35], they have been widely used as natural food additives and accepted by the consumers all over the world. Commercial orange EOs are mainly prepared by the cold pressing method. They consist of volatile components such as terpenes along with some nonvolatile waxy materials, carotenoids, and flavonoids. A hydrodistillation method to prepare orange EO was used in the laboratory on a small scale. Molecular distillation is very useful to separate thermally sensitive EOs and is extensively used in the flavor and fragrance industry. In our study, molecular distillation was used to remove undesired pigments and waxes from the cold pressed EO to provide EO fractions which are potentially beneficial for use as chemotherapy agents. If we change the parameters of molecular distillation, different EO fractions will be obtained. In the future, we will test more EO fractions to find ones with even better anticancer activity. This method can also be applied to other essential oils.

Essential oils are among of the most valuable plant products used in medicine and complementary treatment strategies [36]. However, the beneficial role of NOEO and its constituents in cancer treatment is not clear yet. In order to identify the possible anticancer components of navel orange oil, seven major components were tested. They were observed to have good anticancer activity, with IC_50_ values ranging from 141 to 22 μg/mL. The seven components were d-limonene, which is a representative monoterpene in many citrus species, linalool, 3-carene, α-terpineol, decanal, citral and α-pinene. The IC_50_ values of these seven compounds were all higher than that of NOEO, suggesting that other non-tested constituents in the essential oil might be responsible for the activity of the oil. NOEO prepared by molecular distillation could be considered a potential source of new active anticancer agents. In order to appraise the practical value of this possible therapeutic application, further evaluation should be carried out to clarify the mechanism of action of the seven bioactive components.

## 4. Materials and Methods

### 4.1. Plant Material

Gannan Newhall navel oranges were used as experiment material and collected in January 2015 from Ganzhou City in Jiangxi Province, China.

### 4.2. Chemicals

Linalool, 3-carene, α-terpineol, decanal, citral, d-limonene, α-pinene and *n*-alkanes (C8–C20) were purchased from Sigma-Aldrich (St. Louis, MO, USA). 3-(4,5-Dimethyl-2-thiazolyl)-2,5-diphenyl-2*H*-tetrazolium bromide (MTT) was purchased from Biosharp (Hefei, China).

### 4.3. Preparation of Navel Orange Essential Oil Sample

#### 4.3.1. Cold Pressed Orange Oil Preparation

The recovery of orange oil by FOMESA extractor (Model 391, Food Machinery Espanola, S.A., Valencia, Spain) [37] was carried out during simultaneous extraction of juice and oil. The crude oil emulsion was passed through a 20 mesh shaker screen, and then separated by three-stage centrifugation using three phase disc stack centrifuge separators to achieve complete separation of the oil phase.

#### 4.3.2. Sample Preparation

The purified orange oil sample (NOEO) was obtained by molecular distillation of cold pressed oil using a wiped-film molecular distillation apparatus (Pope 2 inch wiped-film molecular evaporator, Pope Scientific Inc., Saukville, WI, USA) under the following conditions: the evaporation temperature and operation pressure were 60 °C and 1.33 KPa, respectively. The feeding rate was 2.5 mL/min and the speed of the wiper was 350 r/min. The final orange oil sample (NOEO) was obtained from the light phase outlet.

### 4.4. GC-MS Analyses

The constituents of orange oil sample were analyzed using an Agilent 7890B gas chromatograph coupled with an Agilent mass spectrometer detector (Agilent Technologies, Santa Clara, CA, USA). The GC was equipped with a DB-5 column (30.00 m × 0.25 mm × 0.25 µm). Mass spectra were obtained by electron ionization (EI) at 70 eV. The injector and detector were operated at 250 °C and 300 °C, respectively. The temperature program was 80 °C for 4 min, and then increased at 5 °C/min to 250 °C and held constant for 10 min. The constituents were identified by comparing their mass spectra with the National Institute of Standards and Technology (NIST) data reference. The retention indices (RI) of the constituents were determined by adding a C8–C20 *n*-alkanes mixture to the essential oil before injecting in the GC-MS equipment and analyzing it under the same conditions described above.

### 4.5. Free Radical-Scavenging Capacity

The free radical-scavenging activity of NOEO was measured using the stable radical 2,2-diphenyl-1-picrylhydrazyl (DPPH) assay. The analysis was performed in microplates by adding different concentrations of essential oil (1 mL) to a methanolic DPPH solution (3 mL, 0.1 mmol/L). The absorbance at 517 nm was read in an ultraviolet–visible spectrophotometer (UH-5300, Hitachi, Tokyo, Japan). The absorbance of DPPH without NOEO (control sample) was used as the baseline measurement.

The scavenging activity was expressed as the 50% inhibitory concentration (IC_50_), which was defined as the sample concentration (μg/mL) necessary to inhibit DPPH radical activity by 50% after a 1-h incubation. These experiments were performed in triplicate (three independent experiments and three technical replicates for each experiment), and the results are expressed as the mean ± standard deviation.

### 4.6. ABTS Radical-Scavenging Assay

The antioxidant activity of NOEO was measured by ABTS assay according to the protocols of an ABTS kit (S0119, Beyotime Biotechnology, Shanghai, China). The absorbance at 734 nm was then read in a microplate reader (Synergy H1MF, BioTek, Winooski, VT, USA). The results are expressed as the mean ± standard deviation.

### 4.7. Cell Culture

The human lung cancer cell line A549 and prostate cancer cell line 22RV1 were purchased from the cell bank of the Chinese Academy of Sciences (Shanghai, China). A549 cells were cultured in Dulbecco’s modified Eagle’s medium supplemented with 10% fetal bovine serum (FBS) and 1% penicillin streptomycin (C0222, Beyotime Biotechnology). 22RV1 cells were cultured in RPMI-1640 medium containing 10% fetal bovine serum (FBS) and 1% penicillin streptomycin. All cells were cultivated in a 5% CO_2_ atmosphere at 37 °C.

### 4.8. MTT Assay

The NOEO and single constituents of essential oil were dissolved in dimethyl sulfoxide (DMSO). The tested cells (1 × 10^4^ cells per well) were treated with orange oil at different concentrations (6.25–200 μg/mL) in a 96-well culture plate for 24, 48, 72 h. The cells were incubated with 20 μL of MTT reagent (0.5 mg/mL) at 37 °C for 4 h, and then washed three times with 1× phosphate buffered saline (PBS). DMSO (150 μL) was added and the absorbance of each sample was recorded at 570 nm. The experiments were repeated three times and each experiment had three replicate wells.

### 4.9. Annexin-V FITC/PI Assay

A549 cells were treated with NOEO for 24 h and measured by an Annexin-V FITC/PI kit (C1062, Beyotime Biotechnology). Samples were analyzed by flow cytometry (Accuri C6, Becton, Dickinson and Company, Franklin Lakes, NJ, USA). At least 20,000 events per sample were acquired.

### 4.10. Statistical Analysis

Data are expressed as mean values ± standard deviations (SD) from at least three determinations. IC_50_ values of anti-proliferative were assessed by logarithmic regression curves with 95% confident limits. The statistical differences of experimental data were evaluated by Duncan’s multiple range tests (SPSS 18.0 software, SPSS Inc., Chicago, IL, USA). The *p*-values of <0.05 were considered to be significant.

## 5. Conclusions

In this study, an essential oil (NOEO) was obtained by molecular distillation of cold-pressed navel orange essential oil. NOEO displayed good anti-oxidation and anti-cancer activity. We described a novel method to prepare essential oil distillation fractions that can be used as a potential chemotherapeutic agent for the treatment of lung cancer and prostate cancer.

## Figures and Tables

**Table 1:** Chemical composition of navel orange essential oil (NOEO).

No.	Retention Time (min)	RI ^a^	Compounds	Composition (%)
**1**	4.18	934	α-Pinene	1.2
**2**	5.01	974	Sabinene	0.7
**3**	5.31	988	β-Myrcene	1.5
**4**	5.89	1012	3-Carene	0.3
**5**	6.90	1049	Limonene	74.6
**6**	8.38	1103	Linalool	0.8
**7**	9.14	1129	*trans*-*p*-Mentha-2,8-dien-1-ol	1.4
**8**	9.44	1139	Limonene 1,2-epoxide	3.5
**9**	9.57	1144	*cis*-*p*-Mentha-2,8-dien-1-ol	3.2
**10**	9.88	1154	Dihydrocarveol	0.1
**11**	10.73	1183	Citral	0.4
**12**	11.00	1192	Pinocarveol	0.3
**13**	11.24	1201	α-Terpineol	0.8
**14**	11.35	1205	*p*-Mentha-1,8-dien-7-ol	1.4
**15**	11.48	1209	Decanal	0.1
**16**	11.97	1226	(*E*)-Carveol	2.4
**17**	12.35	1240	(*Z*)-Carveol	1.4
**18**	12.70	1252	Carvone	2.4
**19**	13.55	1281	Perillaldehyde	0.3
**20**	13.95	1295	*p*-Mentha-1(7),8(10)-dien-9-ol	0.3
**21**	14.20	1304	Perillyl alcohol	0.3
**22**	16.22	1379	α-Copaene	0.2
**23**	16.53	1390	β-Cubebene	0.1
**24**	19.24	1496	Valencene	0.2
Total				97.9

RI ^a^, retention indices determined on DB-5 column, using the homologous series of *n*-alkanes (C8–C20).

**Table 2:** IC_50_ values of linalool, 3-carene, α-terpineol, decanal, citral, d-limonene, α-pinene and NOEO on proliferation of A549 cells.

Compounds	IC_50_ (μg/mL) ^1^
Linalool	141.76 ± 14.59 ^2a^
3-Carene	70.80 ± 8.24 ^2b^
α-Terpineol	51.37 ± 4.92 ^2c^
Decanal	37.10 ± 2.96 ^2c^
Citral	35.15 ± 2.38 ^2c^
d-Limonene	22.10 ± 1.94 ^2c^
α-Pinene	22.01 ± 1.75 ^2c^
NOEO	17.53 ± 0.95 ^2d^

^1^ IC_50_: compound concentrations that afford a 50% cell growth decrease after 24 h. IC_50_ are the averages of triplicate experiments and represented as mean ± standard deviation; ^2^ Data are presented as mean ± SD, *n* = 3. Means in each column followed by a different letter are significantly different (*p* < 0.05).
